# From pathophysiological aspects towards unravelling the neurobiological background of cognitive deficits

**DOI:** 10.1007/s00406-012-0324-9

**Published:** 2012-05-17

**Authors:** Peter Falkai, Hans-Jürgen Möller

**Affiliations:** 1 Department of Psychiatry and Psychotherapy, University of Göttingen, Von-Siebold-Str. 5, 37075 Göttingen, Germany; 2 Department of Psychiatry and Psychotherapy, Ludwigs-Maximilians-University Munich, Nussbaumstr. 7, 80336 Munich, Germany

Evidence-based medicine is supposed to be a conclusive summary of available empirical knowledge on certain medical issues and as such serving as the basis of guidelines and treatment recommendations. Meant to be self-explaining, the term actually appears to be crucial. It seems inconsistent and inconclusive since recommendations of single countries/institutions are based on different evidence measures—some derive evidence from meta-analyses, whereas others exclusively follow randomised controlled studies. In a philosophical side trip, Möller [1] critically highlights these internationally different measures upon which grading of evidence is levelled and claims a uniform evidence grading.

In mild major, minor or subsyndromal depression, the total score of the Hamilton Depression Rating Scale (HAMD) so far is regarded as ‘gold standard’ in evaluating efficacy of antidepressant treatment. Nevertheless, in comparing the full scales HAMD17 and the Inventory of Depressive Symptomatology (IDS-C28) plus diverse unidimensional subcales in a randomised, placebo-controlled trial in a representative patient sample, Helmreich et al. [2] found the full scales outmatching subscales and the IDS to be superior to the HAMD in detecting symptom changes. Although the time- and cost-saving subscales are able to judge drug therapy outcomes, they fail in covering all depression facets for which reason the cost-to-benefit ratio should be carefully assessed in the decision against using full scales.

In the pathophysiology of depression, glutamate plays an important role. Exploring the association of major depressive disorder with some single nucleotide polymorphisms within the glutamatergic AMPA receptor subunits *GRIA1*, *GRIA2* and *GRIA4*, Chiesa et al. [3] in a relatively small patient sample genotyped Korean inpatients with major depressive disorder in order to possibly predict clinical outcomes. Although no association between alleles, genotypes and haplotypes under investigation plus clinical and demographical variables was detectable, the group found evidence for a possible association between the single nucleotide polymorphisms rs4302506, rs4403097 within GRIA2 and age of depression onset. Anyhow, these results should be substantiated in larger cohorts.

Although an association between affective disorders and the metabolic syndrome has been suspected, past studies did not bear clear results in this respect. Hence, Kahl et al. [4] have examined the prevalence of the metabolic syndrome in 230 male and female inpatients with unipolar major depressive disorder compared to 1,673 controls from primary care from a similar northern German region using the AHA/NHBLI criteria to determine rate and each single criterion of the metabolic syndrome. The inpatient group showed a 2.4× as high prevalence of the metabolic syndrome versus data from controls and elevations for fasting glucose and triglycerides in both genders plus waist circumference in women. Men in both study groups again had higher rates of increased fasting glucose and triglycerides than women. Furthermore, the authors found an association between severity of depression and metabolic syndrome in inpatients and postulate better treatment interventions for metabolic abnormalities and screening for physical health conditions in patients with major depression. Not only due to seasonality, 25-hydroxyvitamin D and hormone levels may be important in the development of depression. To investigate whether the association between depression and low serum 25-hydroxyvitamin D (25(OH)D) and elevated serum parathyroid hormone (PTH) in clinical settings also applies to individuals beyond, Jaddou et al. [5] in a Jordanian national population-based household sample screened 4,002 individuals for depression with the DASS21 depression scale and measured serum concentrations of 25(OH)D and PTH by radioimmunoassay. They found a statistically significant relationship between serum 25(OH)D and depression, but not for PTH. Since the decrease in the risk of depression among participants became significant with serum 25(OH)D levels higher than 42.3 ng/ml, reaching the desirable level might be helpful in preventing and treating depression.

Cognitive deficits play a major role in psychiatric disorders like depression and schizophrenia, but also in neurodegenerative diseases. In an fMRI study in healthy probands, Voss et al. [6] investigated the contribution of the muscarinic receptor system on cognitive performance via applying scopolamine and found hypoactivations in parietal, occipital and cerebellar areas plus in frontal and prefrontal areas. Their results speak for a contribution of muscarinic transmission on cerebral activation. Cholinesterase inhibitors are widely used in the therapy of cognitive deficits in Alzheimer’ disease. Since diffusion tensor imaging (DTI) has shown a decline of fractional anisotropy as marker of fibre tract integrity in Alzheimer’s disease, Likitjaroen et al. [7] compared the longitudinal course of white matter microstructural changes in patients with Alzheimer’s disease and healthy elderly controls and evaluated treatment effects with the cholinesterase inhibitor galantamine versus placebo in the patient group. Despite a seemingly significant decline of fractional anisotropy in certain brain regions after 6-month follow-up, at 12-month follow-up, the impact of galantamine was only limited and between-group differences across patients and controls were no longer detectable.

DTI also was used by Konrad et al. [8] to investigate 12 white matter regions-of-interest within the attentional network of adult patients with attention-deficit/hyperactivity disorder (ADHD) compared to healthy controls. Additionally, all subjects underwent clinical interviews, rating scales and neuropsychological tests. Their findings support a disturbed frontostriatal structural connectivity and an involvement of the left temporal white matter to be responsible for attentional deficits.

In all, cognitive deficits are core symptoms of psychiatric disorders and responsible for an unfavourable outcome. The investigation of the neurobiological basis of these symptoms is needed to develop new regenerative treatment strategies in psychiatric and neurodegenerative diseases.
